# Controlling stem cell fate using cold atmospheric plasma

**DOI:** 10.1186/s13287-020-01886-2

**Published:** 2020-08-26

**Authors:** Fei Tan, Yin Fang, Liwei Zhu, Mohamed Al-Rubeai

**Affiliations:** 1 Department of ORL-HNS, Affiliated East Hospital of Tongji University, Shanghai, China; 2 School of Medicine and Institute for Advanced Study, Tongji University, Shanghai, China; 3 The Royal College of Surgeons of England, London, UK; 4 School of Chemical and Bioprocess Engineering, and Conway Institute of Biomolecular and Biomedical Research, University College Dublin, Dublin, Ireland

**Keywords:** Atmospheric plasma, Stem cell, Cell attachment, Cell proliferation, Cell differentiation, Plasma medicine, Regenerative medicine, Cell death, Tissue engineering, Cold plasma, Non-thermal plasma, Stem cell niche, Surface modification, Scaffold, Extracellular matrix, Plasma-activated medium

## Abstract

The stem cell is the foundation of regenerative medicine and tissue engineering. Regulating specific stem cell fate, such as cell attachment, proliferation, differentiation, and even death, undergoes continuous development. Cold atmospheric plasma (CAP), the core technology of plasma medicine, is attracting tremendous attention due to its ability and versatility to manipulate various types of cells, including stem cells. Specifically, the direct and indirect applications of CAP in controlling cell fate are best exemplified by upfront irradiation of the stem cells and modification of the stem cell niche, respectively. This review will describe the recent advances in various CAP strategies, both direct and indirect, and their influence on the fate of healthy and cancer stem cells. Particular emphasis will be placed on the mechanism of connecting the physical and chemical cues carried by the plasma and biological changes presented by the cells, especially at the transcriptomic level. The ultimate goal is to exploit CAP’s potential in regenerative medicine.

## Cold atmospheric plasma

**Fig. 1:**
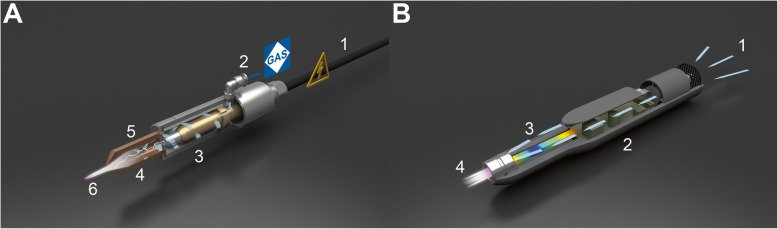
Schematics of atmospheric plasma generated using different technologies. **a** Pulsed atmospheric arc (PAA) technology. (1) High voltage cable, (2) gas inlet, (3) inner electrode (anode), (4) electrical arc, (5) nozzle (cathode), and (6) down-stream plasma. **b** Piezoelectric direct discharge (PDD) technology. (1) air inlet, (2) open piezoelectric transformer, (3) plasma generator, and (4) down-stream plasma

In physics, plasma is the so-called fourth state of matter consisting of roughly equal numbers of positively and negatively charged particles. It could be produced typically at very high temperature or at low pressures and acquired either naturally or artificially. Cold atmospheric plasma (CAP) is a partially ionized gas generated at atmospheric pressure and operates under room temperature. This term is sometimes interchangeable with low-temperature plasma (LTP), non-thermal atmospheric plasma (NTAP), and non-equilibrium atmospheric plasma (NAP). Various technologies have been used to generate atmospheric plasma (Fig. 1), including, but are not limited to, pulsed atmospheric arc (PAA) technology (Fig. 1a) and piezoelectric direct discharge (PDD) technology (Fig. 1b), each with distinct advantages and disadvantages. A detailed comparison between these two technologies and their medical applicability can be found in Table 1.

**Table 1:** Comparison between the pulsed atmospheric arc (PAA) technology and piezoelectric direct discharge (PDD) technology used to generate atmospheric plasma

	PAA technology	PDD technology
Mechanism	Based on the ignition of an electric arc between two electrodes by means of pulsed high voltage; gas flow is ionized as it passes close to the arc, which creates a plasma jet of highly reactive gaseous species	Based on the direct electrical discharge at an open piezoelectric transformer; this dissociates and ionizes the surrounding process gas, which is typically ambient air
Pros	High process speed; adjustable plasma temperature; long-term stability	Very compact; high efficiency; ambient temperature
Cons	Requires automation; need to avoid undesired thermal over-treatment; operate at high power consumption,	Relatively low power output; potential electrical hazard to user due to proximity
Medical applicability	Indirect applications: cell or tissue-resident niche; surgical implant	Direct applications: cells, tissues, and animal or human organs

**Fig. 2:**
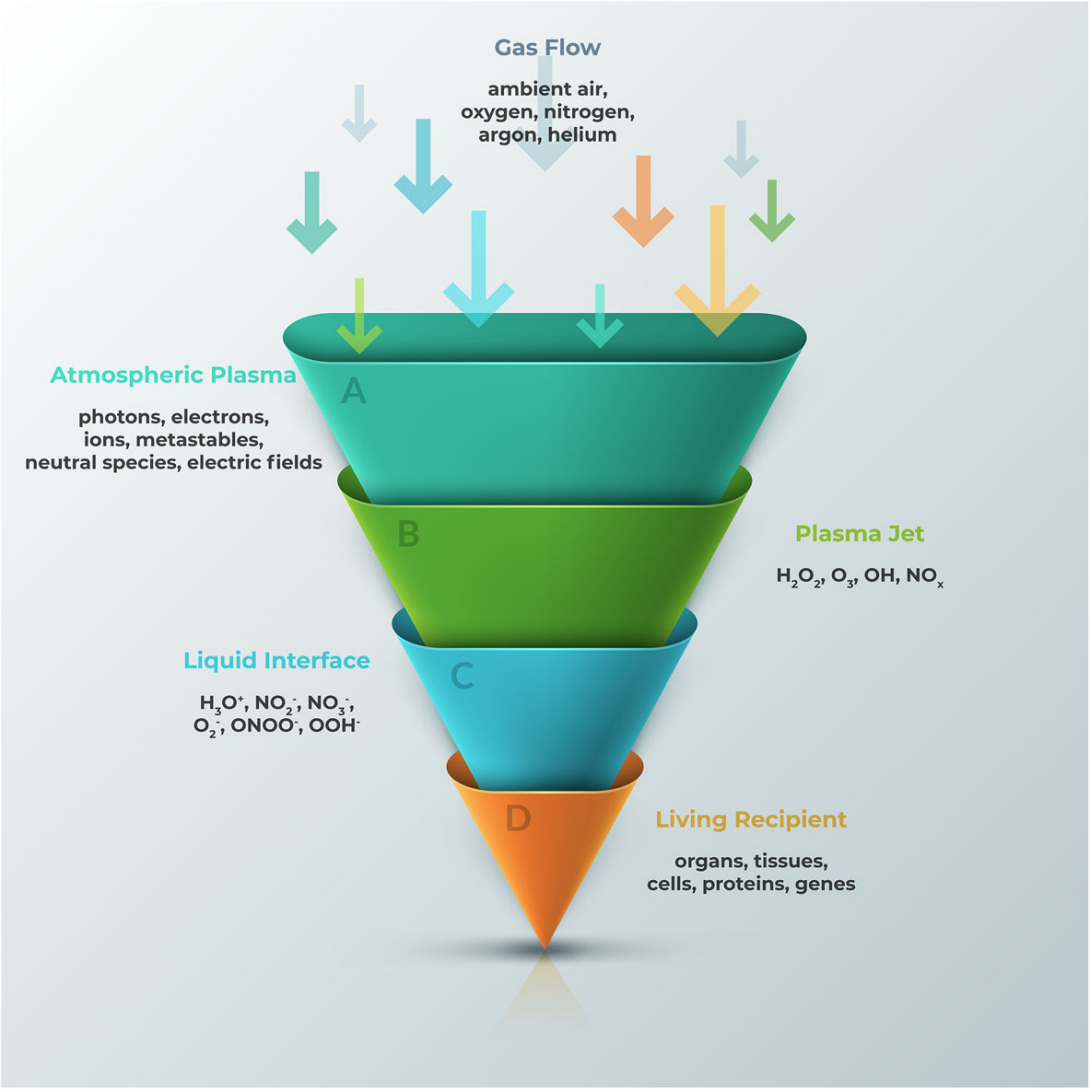
Hierarchical delivery of plasma-derived reactive species towards stem cells and stem cell niche. (A) Atmospheric plasma phase, and its main components. (B) Plasma jet, and the reactive species contained in its plume. If CAP is used as an indirect approach to activate a stem cell niche, the entire process occurs when plasma jet interacts with the solid interface of a niche. However, if CAP is used to directly stimulate a living recipient, it propagates through a liquid phase first. (C) Liquid interface, and various RONS created upon diffusion through it. (D) The biological effect of plasma penetrates across various levels

The key components of CAP include reactive oxygen and nitrogen species (RONS), ions and electrons, and UV photons [1]. The composition and concentration of these components can be tuned and programmed for various applications, especially biomedical ones. In order for CAP to directly influence living recipients, such as cells, the plasma-derived reactive species need to pass various non-biological and biological phases (Fig. 2). Once the atmospheric plasma exits the nozzle and forms a jet, it contains reactive species including H_2_O_2_, O_3_, OH, and NO_x_. Then, they react with the extracellular fluid to form further reactive species including H_3_O^+^, NO_2_^−^, NO_3_^−^, O_2_^−^, ONOO^−^, and OOH^−^. Neutral diffusion of these RONS finally triggers intracellular events.

## Plasma medicine

Plasma medicine is an emerging interdisciplinary field incorporating physics, chemistry, life science, and medicine. It has infiltrated several medical specialties with promising clinical or preclinical potentials [2]. In dermatology, CAP can functionally promote wound healing and cosmetically achieve skin rejuvenation [3]. In microbiology, applications of CAP are exemplified by eradication of microbial biofilm and disinfection of contaminated tissue [4]. In oncology, CAP induces senescence, detachment, apoptosis, and necrosis of tumour cells, demonstrating not only cancer selectivity but also synergy with existing anti-cancer treatment [5].

**Fig. 3:**
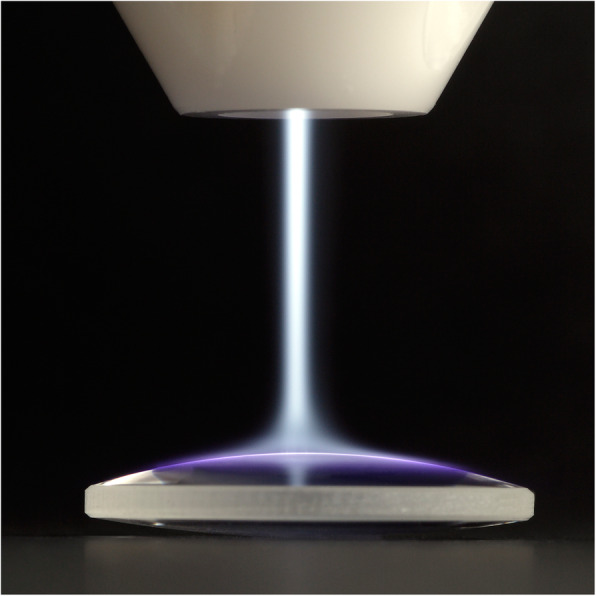
Interfacial modification of an artificial stem cell niche using CAP. Image was taken when using a handheld plasma device based on piezoelectric direct discharge (PDD) technology. The gaseous plasma jet can be seen exiting the nozzle and spreading over the target surface

In addition to these direct plasma treatments on the living tissue, indirect treatments of the biological environment or the niche around the tissues or cells also have attracted extensive research. One example is surface modification of an artificial cell niche, e.g. tissue engineered scaffold or surgical implants (Fig. 3). The other modern example is the plasma-activated medium (PAM) [6]. PAM is inspired by the scientific basis of plasma-liquid interactions in which the reactive chemistry of CAP is transferred and retained prior to a chain of biological events. It can potentially be injected to treat deep cancers or cancers that have spread over large areas. Whether used directly or indirectly, CAP is at the heart of advancing plasma medicine.

## Stem cell niche and control of stem cell fate

**Fig. 4:**
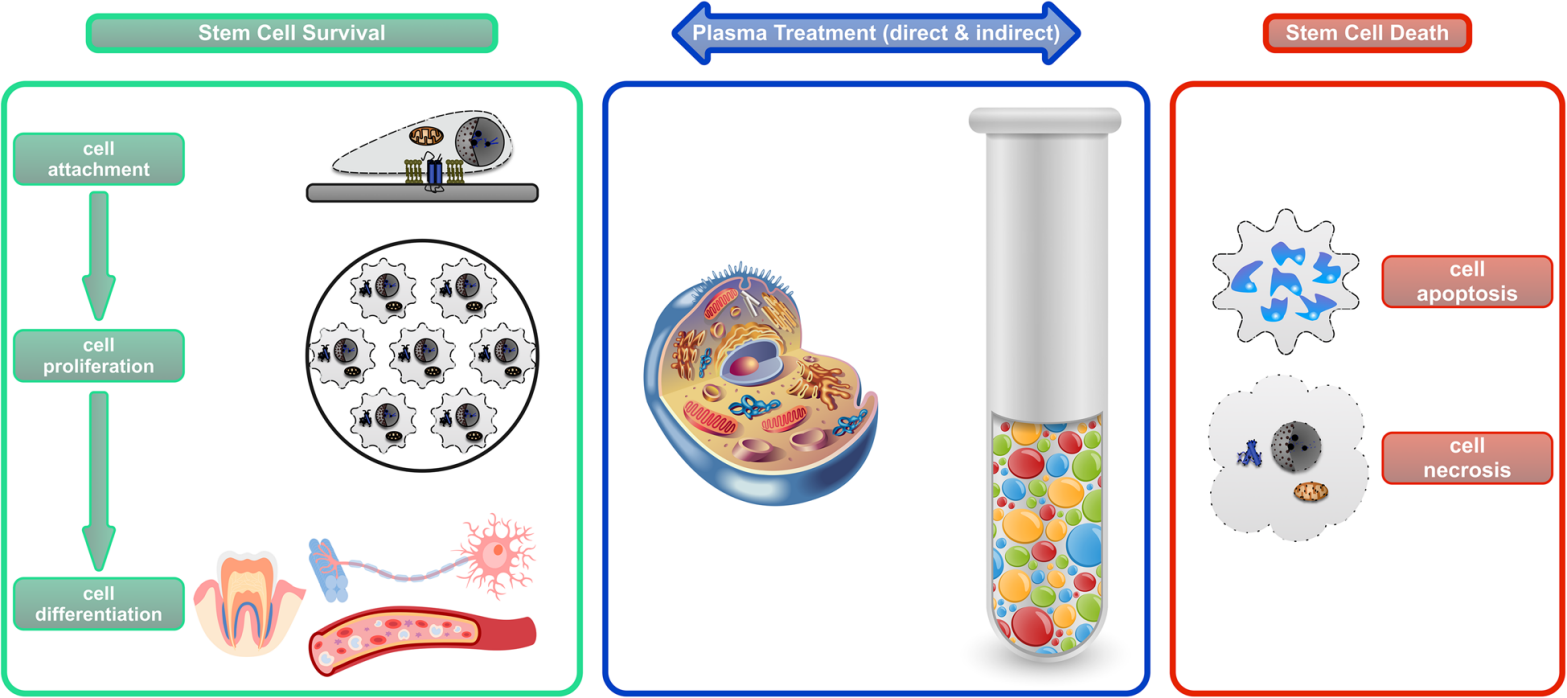
The influence of plasma treatment on the survival and death of stem cells. The box in the middle depicts the two main strategies of plasma treatment, directly on the cells and indirectly on the cell niche; the box on the left highlights the main events during stem cell survival, whereas the box on the right covers the main forms of stem cell death

Stem cells are revolutionizing modern medicine mainly because of their clinical potential to promote the relief, repair, and regrowth response of diseased or damaged tissue. Stem cells exert these regenerative functions through their dynamic interaction with the surrounding microenvironment or the niche in which they reside [7]. The resultant homeostasis determines the fate of stem cells, either survival or death (Fig. 4). Therefore, possible fate options of stem cells include, but are not limited to, attachment (anchoring to the niche), proliferation (self-renewal), differentiation (lineage specification), and death (active, programmed, or passive, accidental) [8].

Conventional methods of controlling the fate of stem cells are mostly physical and chemical modulation of the stem cell niche. Adjustable physical and mechanical stimuli include strain, shear stress, matrix rigidity, and topography [9] whereas the tuneable chemical cues include exogenous growth factors, cytokines, and various small molecules [10]. Recent advances in biomaterial research have attempted to partially or entirely replace the stem cell microenvironment with engineered synthetic materials [11]. This has evolved from simple two-dimensional (2D) cultures to complex three-dimensional (3D) scaffolds. No matter the approach, as mentioned above, is applied, their effects are considered ‘indirect’ as they universally target the niche in which stem cells inhabit.

In comparison, CAP is a unique technology to regulate and direct stem cell fate due to its excellent versatility. Not only can CAP indirectly control cell fate by modifying the cell-resident niche, it can also directly influence cell fate by stimulating stem cells in close contact (Fig. 4). This mini-review presents an up-to-date view of the versatility of CAP in controlling stem cell fate, highlighting a novel strategy for regenerative medicine and other applications.

## Enhancing stem cell attachment using CAP

The cell-niche adhesion is crucial for stem cell survival and is dynamically adapted. CAP has recently been proven capable of improving stem cell’s attachment to the natural niche and artificial extracellular matrix (ECM). This enhancement is mostly indirect, as CAP modifies the cell-facing niche or ECM (Table 2).

**Table 2:** Enhancing stem cell attachment and promoting stem cell proliferation using CAP. (*ASCs* adipose-derived stem cells, *ECM* extracellular matrix, *ESCs* embryonic stem cells, *HA* hydroxyapatite, *HSCs* haematopoietic stem cells, *MSCs* mesenchymal stem cells, *NO* nitric oxide, *NSCs* neural stem cells, *NT* neurotrophin, *PCL* polycaprolactone, *PS* polystyrene, *PSCs* pluripotent stem cells, *PU* polyurethane, *USSCs* unrestricted somatic stem cells)

	Mode of action	Novelty	Ref
Enhancing stem cell attachment	Mostly indirect by surface-modifying the stem cell niche or ECM	Chemical modification turns PS hydrophilic and supports optimal PSC attachment and long-term self-renewal	[12]
		CAP also endows mechanical improvement of surface, which enhances the adhesion and spreading of MSCs	[13]
		Nitrogen plasma is better than oxygen and air plasma in improving MSCs attachment on gelatin scaffolds	[14]
		CAP modification of PU scaffolds results in differential increments of cell attachment for ESCs and NSCs	[15]
		Nanoscale, rather than microscale, PCL scaffolds attract larger benefits from CAP treatment for MSC adhesion	[16]
		CAP-modified cochlear implant electrode array surface enables colonization of NT-secreting ASCs	[17]
Promoting stem cell proliferation	Either indirect stimulation of cell niche or direct exposure of stem cells	Oxygen plasma is better than argon plasma in promoting USSCs proliferation on PS surface	[18]
		CAP-treated gelatin films support better MSCs proliferation, with the optimal hydrophilicity at 27–32°	[19]
		Better MSCs proliferation on HA surface is due to faster progression of cell cycle at a transcriptomic level	[20]
		CAP increases ASCs’ proliferation by 60% while maintaining cellular stemness, through NO upregulation	[21]
		CAP increases MSCs’ and HSCs’ proliferation by twofold, and activates relevant gene expression	[22]
		An epigenetic study in which CAP upregulates genes for cytokines, chemokines but downregulates apoptosis	[23]

Due to its intrinsic mechanism, CAP is well known to introduce chemical changes to the cell culture surface. One of the essential parameters of surface chemistry is surface energy, which is commonly reflected by wettability. Ueda et al. discovered that human pluripotent stem cells (PSCs) adhered poorly to hydrophobic cell culture dishes [12]. However, after turning the surface hydrophilic using CAP treatment, the chemically modified polystyrene (PS) surface could support optimal PSC attachment and long-term self-renewal. Unlike the traditional understanding that CAP solely induces chemical modification, a new theory by Yang et al. suggesting that CAP also alters other surface properties has gained popularity [13]. In their work, CAP treatment of the polymeric substrate also changed the topography and elasticity at the nanoscale level. The predominant mechanical effect enhanced the adhesion and spreading of human mesenchymal stem cells (MSCs) through good focal adhesions.

In addition to the 2D surfaces (e.g. laboratory cell culture system), 3D interfaces (e.g. tissue engineering scaffold) are also conveniently processable using CAP. The extent of enhancement depends on several factors including, but are not limited to, plasma parameters, type of stem cells, and the scale of the scaffold. Firstly, the quantity of bone marrow-derived MSCs (BM-MSCs) attached to CAP-treated gelatin scaffolds was significantly increased compared to untreated specimens [14]. Also, among the three types of working gas used (nitrogen, oxygen, and air), nitrogen plasma provided the best MSC attachment, owing to the N-containing functional groups generated on the surface. Secondly, surface modification of polyurethane (PU) scaffold using CAP led to increased cell attachment [15]. However, the increments were substantially different for human embryonic stem cells (ESCs) and rat postnatal neural stem cells (NSCs). Finally, Sankar et al. subjected polycaprolactone (PCL) fibrous scaffolds with nano-, micro-, and multiscale to CAP treatment. They discovered that the nanofibers demonstrated most remarkable MSC adhesion, spreading, and elongation [16].

The relevant research priority has shifted in the last several years; the emerging applications use genetically modified stem cells. In the study performed by Schendzielorz et al., CAP-assisted hydrophilization of cochlear implant electrode array surfaces enabled firm colonization of human adipose-derived stem cells (ASCs) that are known to secrete neurotrophins [17]. This long-term delivery of neurotrophic factors has profound clinical potential for treating inner ear diseases and neurotology conditions.

In summary, CAP can enhance stem cell attachment to their adjacent environment, mostly by activating the support surfaces. The mechanism of enhanced cell attachment is most likely due to the improved physicochemical changes of the surface, which enhance the expression of adhesion molecules, thus leading to favourable cell attachment.

## Promoting stem cell proliferation using CAP

The continued self-renewal and proliferation drives the functionality of stem cells in regenerative medicine. CAP can accelerate the growth of stem cells while maintaining their stemness. This improvement in cell proliferation is achieved through either indirect stimulation of cell-resident niche or direct exposure of stem cells (Table 2).

On the one hand, CAP can speed up cell growth by activating the cell-niche interface, regardless of the interface material. These materials range from regular cell culture plastic, such as PS [18], bioceramic, such as hydroxyapatite (HA) [20], to ECM-based protein, such as gelatin [19]. However, CAP-induced optimization of the surface chemistry seems to be the central cause for the cellular benefit. Biazar and colleagues demonstrated better proliferation of unrestricted somatic stem cells (USSCs) on the PS surfaces treated with oxygen or argon CAP than on the untreated samples [18]. Also, oxygen plasma provided more significant advantage than argon plasma, not just because of higher ensuing hydrophilicity, but also partially due to topographical changes, i.e. larger surface nano-roughness. In Prasertsung’s study, the proliferation of BM-MSCs was significantly higher on the CAP-treated gelatin films than on the unmodified ones [19]. This observation was confirmed by shorter population doubling time and a higher growth rate of cells. Furthermore, the optimal water contact angle, a marker for hydrophilicity and surface energy, was observed at 27 to 32 degrees. Tan et al. concurred with these results and further explored their cellular and genetic mechanism [20]. In order to ascertain the actual growth rate of human MSCs on the HA samples, flow cytometry was used to analyse the dynamic distribution of cell cycle phases. It was suggested that the favourable cell proliferation on plasma-activated surfaces was a result of faster progression of the cell cycle, likely mediated by preferential expression of focal adhesion kinase (FAK). This was one of the first studies revealing CAP-regulated biological benefits at a large-scale transcriptomic level.

On the other hand, CAP can enhance cell proliferation by irradiating the stem cells in situ. This possibility was exemplified by a series of step-wise investigations conducted by Park and her team [21–23]. In 2016, they showed that helium-based CAP increased proliferation of ASCs by nearly 60% after 3 days of incubation, compared with untreated cells [21]. Plasma-exposed ASCs maintained cellular stemness and their ability to differentiate into adipocytes, without demonstrating cellular senescence. In addition, Akt, ERK1/2, and their downstream NF-κB were all activated in ASCs after plasma exposure. These results collectively showed that nitric oxide (NO) rather than reactive oxygen species (ROS) is responsible for the increased proliferation of ASCs following CAP exposure. In 2019, Park et al. extended their stem cell choice from ASCs to various mesoderm-derived human adult stem cells [22]. They discovered that CAP increased proliferation of BM-MSCs and haematopoietic stem cells (HSCs) by almost twofold. Furthermore, CAP treatment of these stem cells also activated expression of stem cell-specific surface markers, such as CD44 and CD105, and common pluripotent genes for stemness, such as *Oct4*, *Sox2*, and *Nanog*. This year, the South Korean research group took the relevant work one step further by exploring the epigenetic mechanism by which CAP activates the proliferation of stem cells [23]. After examining the entire genome expression profiles of ASCs, they found that CAP upregulated genes for cytokines, chemokines, and growth factors, but downregulated the genes of intrinsic apoptotic pathways. Once again, plasma-induced epigenetic modifications at both mRNA and protein levels mainly relied on the NO generated from CAP.

In brief summary, CAP can promote stem cell proliferation directly and indirectly. These are realized by either exposing the stem cells to plasma or activating the cell-niche interface. The mechanism of accelerated cell proliferation is a result of multilevel events, including faster cell cycle at the cellular level, activated stem cell-specific markers at the protein level, and advantageous deregulation of genes at the transcriptomic level.

## Inducing stem cell differentiation using CAP

One of the defining features of stem cells is their capability to differentiate into various types of more specialized cells. This process usually involves a switch from proliferation to specialization, as well as alterations in cellular morphology, function, and fate. CAP can induce and/or boost the lineage-specific differentiation of stem cells into hard tissues or soft tissues, through direct and/or indirect non-thermal treatment (Fig. 4). Cell differentiation induced by CAP-assisted coating deposition and drug delivery has been discussed elsewhere and, therefore, will not be discussed here.

CAP can induce differentiation of stem cells into hard tissues, such as bone, cartilage, and teeth, mostly through modifying cell-resident, tissue-engineered scaffolds (Table 3). Wang et al. achieved the osteogenic differentiation of BM-MSCs in a series of studies [24–26]. Firstly, they surface-modified HA/chitosan scaffolds [24]. A careful investigation found more total and adhesion-mediated protein (e.g. fibronectin and vitronectin) adsorption on the scaffold surfaces, which likely contributed to the enhanced late-stage osteogenic differentiation. Secondly, they combined the micro-scale architecture offered by 3D printed poly(lactic acid) (PLA) scaffolds with the nanoscale roughness and chemical modification provided by CAP, significantly promoting in vitro bone regeneration [25]. Lastly, they studied the cellular infiltration and drug loading in plasma-treated core-shell nanofibers [26]. CAP modification not only increased the pore size of the scaffold resulting in more calcium deposition, but also contributed to significant variations in drug release profiles. Like BM-MSCs, ASCs can also adapt to plasma modification of the scaffolds. Waser-Althaus et al. discovered that the degrees of osteogenic differentiation of ASCs on CAP-treated polyetheretherketone (PEEK) surface depended on plasma power and process gas [27].

**Table 3:** Inducing and enhancing tissue-specific differentiation from stem cells using CAP. (*ASCs* adipose-derived stem cells, *BM-MSCs* bone marrow-derived mesenchymal stem cells, *CJMSCs* conjunctiva-derived mesenchymal stem cells, *MPJs* micro-plasma jets, *NO* nitric oxide, *NSCs* neural stem cells, *PCL* polycaprolactone, *PDL-MSCs* periodontal ligament-isolated mesenchymal stem cells, *PEEK* polyetheretherketone, *PLA* poly(lactic acid), *PU* polyurethane, *RONS* reactive oxygen and nitrogen species)

Target tissue		Stem cells	Novelty	Ref
Hard tissue	Bone	BM-MSCs	CAP provides surface modification of HA/chitosan scaffolds, leading to favourable protein adsorption, and enhanced osteogenic differentiation	[24]
			CAP proves a quick and inexpensive way to modify nanoscale roughness and chemical composition of 3D printed PLA scaffolds with microscale architecture	[25]
			CAP-modified core-shell nanofibers not only has higher surface pore size and osteoinductivity, but also improved drug release kinetics	[26]
		ASCs	Degrees of osteogenic differentiation from ASCs on CAP-treated PEEK surface depend on plasma power and working gas	[27]
	Bone, cartilage		ASCs can be differentiated towards osteogenic and chondrogenic lineages on amine- and carboxyl-modified scaffolds using plasma polymerization, respectively	[28]
			Argon plasma-treated PU scaffolds support not only in vitro osteogenesis and chondrogenesis, but also in vivo tissue ingrowth and angiogenesis	[29]
	Teeth	PDL-MSCS	CAP promotes osteogenic differentiation from PDL-MSCs, although inhibiting cell migration and attenuating cell proliferation	[30]
Soft tissue	Nerve	C17.2- NSCs, primary rat NSCs	MPJs effectively direct in vitro differentiation of NSCs predominantly into neuronal lineage; higher differentiation efficiency than conventional method	[31]
			A detailed experimental protocol with video demonstration of using CAP to accelerate neuronal differentiation rate in a one-step fashion	[32]
		C17.2- NSCs	A gene-level study investigating the mechanism of enhanced and directed differentiation of NSCs by CAP; both extra- and intracellular NO contribute	[33]
		N2a cells	CAP induces neural differentiation through crosstalk between specific RONS cascade and Trk/Ras/ERK signalling pathway	[34]
	Pancreatic islets	CJMSCs	CAP enhances stem cell differentiation into insulin-producing cells on 3D tissue engineering PCL scaffold; new source for diabetes therapy	[35]

Moreover, Griffin et al. examined the role of CAP in guiding ASCs towards both osteogenic and chondrogenic differentiations [28, 29]. On the one hand, ASCs could be differentiated preferentially towards osteogenic and chondrogenic lineages on amine (NH_2_) and carboxyl (COOH) modified scaffolds using CAP, respectively [28]. This chemical group-dependent plasma polymerization method has significant potential for selective stem cell therapy in the regeneration of bone and cartilage. On the other hand, ASCs on the argon CAP-treated polyurethane (PU) scaffolds showed upregulated expression of bone makers (e.g. alkaline phosphatase, collagen I, and osteocalcin) and cartilage markers (e.g. aggrecan and collagen II) [29]. Upon implantation onto the chick chorioallantoic membrane, plasma-treated scaffolds supported higher expression of vascular endothelial growth factor (VEGF) and laminin. These satisfactory in vitro and in vivo results proved CAP to be a simple but efficient tool that can promote ASC skeletal differentiation. Last but not least, CAP can directly induce osteogenic differentiation of stem cells from an unusual source, such as human mesenchymal stem cells isolated from periodontal ligament (PDL-MSCs), expanding its potential for future dental applications [30].

CAP can also induce differentiation of stem cells into forming soft tissues, such as nerves, mostly through directly irradiating NSCs (Table 3). In a series of studies, Xiong et al. achieved neuronal differentiation using murine immortalized neural stem cell line (C17.2-NSCs) and primary rat NSCs in a series of studies [31–33]. Initially, they applied microplasma jets (MPJs), a unique form of CAP, to direct in vitro differentiation of NSCs into the neuronal lineage, with longer neurites and cell bodies constituting a mature neuronal network [31, 32]. The neuronal differentiation rate was around 75% using this one-step approach, which was much higher compared to the standard chemical method. The differentiated NSCs matured and produced mostly cholinergic and motor neuronal progeny. Their preliminary investigation suggested NO to be the main factor in such fate choice. Then, they deepened the investigation of the mechanism of enhanced and directed differentiation of NSCs by CAP, especially the possible signalling pathways stimulated by NO [33]. The exogenous NO in the plasma and the increased synthesis of intracellular NO by inducible nitric oxide synthase (iNOS) after plasma stimulation could be the underlying contributors. Collectively, the extracellular and intracellular NO downregulated *Notch1* and *Id2* and upregulated *Ngn2* and *Ascl1*, thereby activating downstream *NeuroD* expression. As a supplement to the above study, Jang et al. conducted additional upstream and downstream investigations [34]. Specifically, RONS from the plasma phase interacted with reactive species in the extracellular liquid phase to form NO. The extracellular NO reversibly inhibited mitochondrial complex IV, while cytosolic hydrogen peroxide acted as an intracellular messenger to initiate the Trk/Ras/EKR signalling pathway. Thus, this study elucidated the mechanism connecting physicochemical signals from the CAP cascade to the intracellular neuronal differentiation signalling pathway, offering insights into developing of a novel plasma-based treatment for neurological diseases.

Transplantation of pancreatic islet-forming stem cells is a promising therapeutic method for treating diabetes mellitus. However, finding a readily available stem cell source and cell carrier is technically challenging. An interesting work by Nadri et al. combined conjunctiva-derived mesenchymal stem cells (CJMSCs) and PCL scaffold [35]. CAP-treated scaffold supported enhanced differentiation of CJMSCs into insulin-producing cells, with significantly higher insulin release in vitro.

In summary, CAP can induce and/or enhance differentiation of stem cells into various tissues, such as bone, cartilage, and nerve. Again, these could be achieved by either direct exposure or indirect modification. The underlying mechanism of CAP-induced cell differentiation is likely far more complicated than those of CAP-assisted cell attachment and proliferation. But it should be traced back to the complex recipe of RONS generated using highly tunable but sophisticated plasma parameters. Since stem cell attachment, proliferation, and differentiation are a dynamic and seamless chain of events, CAP has the potential to be used as a one-step streamlining tool to facilitate stem cell survival during their fate choices.

## Stimulating cancer stem cell death using CAP

In contrast to cell survival, controlled cell death could also be desired when determining stem cell fate, especially for cancer stem cells. Cancer stem cells (CSCs) are a small subpopulation of cells within tumours, likely initiating cancer recurrence and metastasis. They are capable of self-renewal, differentiation, and tumourigenicity when transplanted into a live host [36]. CSCs are resistant to conventional treatments, such as chemotherapy and radiotherapy. Therefore, efforts are increasing to develop novel anti-cancer treatment modalities targeting the CSCs. CAP proves a promising candidate for such a clinical purpose. The exertion of anti-CSCs using CAP is genuinely versatile, including direct irradiation of cells with or without adjuvant agent [37], plasma-stimulated macrophages [38], and plasma-activated medium (PAM) [39, 40] (Fig. 4).

Although plasma alone can eliminate cancer cells in many preclinical studies using various malignancy models, it has also demonstrated synergy with conventional chemotherapy medications [41]. Adhikari et al. combined CAP with nanotechnology, i.e. silymarin nanoemulsion (SN), for treating melanoma [37]. Co-treatment by SN and CAP increased the cellular toxicity for both melanoma cells and CSCs in a time-dependent manner in vitro and also decreased tumour weight and size in vivo. The p53-mediated apoptosis in these cells was likely activated through inhibition of the HGF/c-MET downstream pathway. Thus, CAP oncotherapy supplemented by SN serves as a new treatment approach for melanoma.

Like direct irradiation of CSCs, indirect CAP treatments, such as co-culture of cancer cells with plasma-activated macrophages, also provide an equivalent anti-cancer function. Kaushik et al. demonstrated a tumour-suppressive role for CAP-stimulated macrophages in solid metastatic cancers that are mediated by epithelial-mesenchymal transition (EMT) [38]. EMT contributes to many malignant behaviours of cancer cells and CSCs, including anti-apoptotic, motile, invasive, and stem-like features. CAP could induce M1/M2 macrophage polarization, with M1 to a greater extent. These M1-polarized macrophages delayed the EMT process in glioma cells, attenuating CSCs maintenance. Therefore, CAP acted as an immune-modulating oncotherapy upregulating pro-inflammatory anti-tumourigenic M1 macrophages.

One of the recent advances of CAP oncotherapy is the usage of PAM, a plasma-treated solution in which the reactive chemistry of gaseous CAP is transferred and retained. Ikeda et al. discovered that PAM selectively induces apoptotic death of cancer cells but not healthy cells. Also, PAM killed both CSCs and non-CSC endometrioid carcinoma and gastric cancer cells [39]. When tested in a mouse xenograft model, PAM also had an anti-cancer effect on CSCs. Therefore, these in vitro and in vivo results supported PAM as a new modality of oncotherapy, targeting various cancer cells, including CSCs. Similar to CSCs, residual undifferentiated human induced PSCs, intended for regenerative medicine and cell transplantation therapy, might also demonstrate tumourigenic potential. Thus, selective elimination of undifferentiated stem cells from a population of differentiated cells before their transplantation is clinically important. Matsumoto et al. achieved this by inducing external oxidative stress using CAP [40]. Undifferentiated PSCs were more sensitive to PAM than PSC-derived differentiated cells. Gene expression and protein assay suggested that hydrogen peroxide and various RONS in PAM was one of the mechanisms underlying PAM-induced selective cell death.

In summary, CAP is a multimodal treatment to suppress or eliminate cancer stem cells and stem cells with tumourigenic potential. Due to the versatility of CAP, the mechanism of plasma-induced CSCs elimination is strategy-dependent.

## Concluding remarks and future directions

How to control stem cell fate is the key question while maximizing its clinical potential in regenerative medicine and cell transplantation therapy. It is difficult for a single technology to regulate all fate options of stem cells, considering the temporal and spatial complexity of cellular events, such as cell attachment, proliferation, differentiation, and death. Cold atmospheric plasma is a promising strategy due to its excellent versatility. Its direct applications, such as irradiating stem cells in close contact, and its indirect applications, such as modifying stem cell niche, have already gained preclinical interests. However, several challenges remain unsolved before this unique tool can be used widely in clinical applications.

Firstly, extensive research is required to elucidate the mechanism of how physicochemical changes induced by plasma are translated into biological benefits. Secondly, although CAP can regulate individual stages of stem cell fate, controlling the transition from one stage to another and balancing the extent of each stage demands further testing. Lastly, CAP-assisted enhancement in stem cell survival and CAP-induced death in cancer stem cells will need to be cell-specific and tissue-specific, thereby providing a customizable therapy based on the exact clinical requirement.

In summary, the innovative applications of CAP in regulating stem cell fate establishes a new frontier in plasma medicine, likely helping to form a next-generation therapy in regenerative medicine.

## Data Availability

The datasets during and/or analysed during the current study are available from the corresponding author on reasonable request.

## Abbreviations

2D: Two-dimensional
3D: Three-dimensional
ASCs: Adipose-derived stem cells
CAP: Cold atmospheric plasma
CSCs: Cancer stem cells
ECM: Extra-cellular matrix
EMT: Epithelial-mesenchymal transition
FAK: Focal adhesion kinase
HA: Hydroxyapatite
HSCs: Haematopoietic stem cells
iNOS: Inducible nitric oxide synthase
LTP: Low-temperature plasma
MPJs: Microplasma jets
MSCs: Mesenchymal stem cells
NAP: Non-equilibrium atmospheric plasma
NO: Nitric oxide
NSCs: Neural stem cells
NTAP: Non-thermal atmospheric plasma
PAA: Pulsed atmospheric arc
PAM: Plasma-activated medium
PCL: Polycaprolactone
PDD: Piezoelectric direct discharge
PEEK: Polyetheretherketone
PLA: Poly(lactic acid)
PS: Polystyrene
PSCs: Pluripotent stem cells
PU: Polyurethane
RONS: Reactive oxygen and nitrogen species
USSCs: Unrestricted somatic stem cells
VEGF: Vascular endothelial growth factor

## References

[1] Adamovich I (2017). The 2017 Plasma Roadmap: low temperature plasma science and technology. J Phys D Appl Phys.

[2] von Woedtke T (2013). Plasmas for medicine. Phys Rep.

[3] Bernhardt T (2019). Plasma medicine: applications of cold atmospheric pressure plasma in dermatology. Oxidative Med Cell Longev.

[4] Gilmore BF (2018). Cold plasmas for biofilm control: opportunities and challenges. Trends Biotechnol.

[5] Hirst AM (2016). Low temperature plasmas as emerging cancer therapeutics: the state of play and thoughts for the future. Tumor Biol.

[6] Kaushik NK (2018). Biological and medical applications of plasma-activated media, water and solutions. Biol Chem.

[7] Ferraro F (2010). Adult stem cels and their niches. Adv Exp Med Biol.

[8] Enver T (2009). Stem cell states, fates, and the rules of attraction. Cell Stem Cell.

[9] Kshitiz (2012). Control of stem cell fate and function by engineering physical microenvironments. Integr Biol (Camb).

[10] Liu K (2016). Chemical modulation of cell fate in stem cell therapeutics and regenerative medicine. Cell Chem Biol.

[11] Bertucci TB, Dai G (2018). Biomaterial engineering for controlling pluripotent stem cell fate. Stem Cells Int.

[12] Ueda Y (2012). Substrates for human pluripotent stem cell cultures in conditioned medium of mesenchymal stem cells. J Biomater Sci Polym Ed.

[13] Yang Y (2012). Effects of topographical and mechanical property alterations induced by oxygen plasma modification on stem cell behavior. ACS Nano.

[14] Prasertsung I (2012). Plasma enhancement of in vitro attachment of rat bone-marrow-derived stem cells on cross-linked gelatin films. J Biomater Sci Polym Ed.

[15] Zanden C (2014). Stem cell responses to plasma surface modified electrospun polyurethane scaffolds. Nanomedicine.

[16] Sankar D (2014). Surface plasma treatment of poly(caprolactone) micro, nano, and multiscale fibrous scaffolds for enhanced osteoconductivity. Tissue Eng Part A.

[17] Schendzielorz P (2014). Plasma-assisted hydrophilization of cochlear implant electrode array surfaces enables adhesion of neurotrophin-secreting cells. ORL J Otorhinolaryngol Relat Spec.

[18] Biazar E (2011). The relationship between cellular adhesion and surface roughness in polystyrene modified by microwave plasma radiation. Int J Nanomedicine.

[19] Prasertsung I (2013). Comparison of the behavior of fibroblast and bone marrow-derived mesenchymal stem cell on nitrogen plasma-treated gelatin films. Mater Sci Eng C Mater Biol Appl.

[20] Tan F (2012). Cellular and transcriptomic analysis of human mesenchymal stem cell response to plasma-activated hydroxyapatite coating. Acta Biomater.

[21] Park J (2016). Non-thermal atmospheric pressure plasma efficiently promotes the proliferation of adipose tissue-derived stem cells by activating NO-response pathways. Sci Rep.

[22] Park J (2019). Non-thermal atmospheric pressure plasma is an excellent tool to activate proliferation in various mesoderm-derived human adult stem cells. Free Radic Biol Med.

[23] Park J (2020). Non-thermal atmospheric pressure plasma induces epigenetic modifications that activate the expression of various cytokines and growth factors in human mesoderm-derived stem cells. Free Radic Biol Med.

[24] Wang M (2014). Design of biomimetic and bioactive cold plasma-modified nanostructured scaffolds for enhanced osteogenic differentiation of bone marrow-derived mesenchymal stem cells. Tissue Eng Part A.

[25] Wang M (2016). Cold atmospheric plasma (CAP) surface nanomodified 3D printed polylactic acid (PLA) scaffolds for bone regeneration. Acta Biomater.

[26] Wang M (2019). Cold atmospheric plasma (CAP)-modified and bioactive protein-loaded core-shell nanofibers for bone tissue engineering applications. Biomater Sci.

[27] Waser-Althaus J (2014). Differentiation of human mesenchymal stem cells on plasma-treated polyetheretherketone. J Mater Sci Mater Med.

[28] Griffin MF (2017). Chemical group-dependent plasma polymerisation preferentially directs adipose stem cell differentiation towards osteogenic or chondrogenic lineages. Acta Biomater.

[29] Griffin MF (2019). Argon plasma modification promotes adipose derived stem cells osteogenic and chondrogenic differentiation on nanocomposite polyurethane scaffolds; implications for skeletal tissue engineering. Mater Sci Eng C Mater Biol Appl.

[30] Miletić M (2013). Effects of non-thermal atmospheric plasma on human periodontal ligament mesenchymal stem cells. J Phys D Appl Phys.

[31] Xiong Z (2014). Selective neuronal differentiation of neural stem cells induced by nanosecond microplasma agitation. Stem Cell Res.

[32] Xiong Z, et al. Nerve stem cell differentiation by a one-step cold atmospheric plasma treatment in vitro. J Vis Exp. 2019;143:e58663. 10.3791/58663 30688309

[33] Zhao SS (2019). Investigation of the mechanism of enhanced and directed differentiation of neural stem cells by an atmospheric plasma jet: a gene-level study. J Appl Phys.

[34] Jang JY (2018). Cold atmospheric plasma (CAP), a novel physicochemical source, induces neural differentiation through cross-talk between the specific RONS cascade and Trk/Ras/ERK signaling pathway. Biomaterials.

[35] Nadri S (2018). Differentiation of conjunctiva mesenchymal stem cells into secreting islet beta cells on plasma treated electrospun nanofibrous scaffold. Artif Cells Nanomed Biotechnol.

[36] Yu Z (2012). Cancer stem cells. Int J Biochem Cell Biol.

[37] Adhikari M (2019). Cold atmospheric plasma and silymarin nanoemulsion synergistically inhibits human melanoma tumorigenesis via targeting HGF/c-MET downstream pathway. Cell Commun Signal.

[38] Kaushik NK, et al. Preventing the solid cancer progression via release of anticancer-cytokines in co-culture with cold plasma-stimulated macrophages. Cancers (Basel). 2019;11(6):842–63. 10.3390/cancers11060842 PMC6628390 31216715

[39] Ikeda JI (2018). Plasma-activated medium (PAM) kills human cancer-initiating cells. Pathol Int.

[40] Matsumoto R (2016). Plasma-activated medium selectively eliminates undifferentiated human induced pluripotent stem cells. Regen Ther.

[41] Dai X (2018). The emerging role of gas plasma in oncotherapy. Trends Biotechnol.

